# The Hospital Nursing Supplement

**Published:** 1896-01-25

**Authors:** 


"The Hospital" Nursing Mirror.

Being the Extra Nursing Supplement of "The Hospital" Newspaper.

[Contributions for this Supplement should be addressed to the Editor, The Hospital, 428, Strand, London, W.C., and should have the word "Nursing" plainly written in left-hand top corner of the envelope.]

### News from the Nursing World.

### THE LATE PRINCE HENRY OF BATTENBERG.

It is with the deepest regret we learn that news of the death of Prince Henry of Battenberg has been received at the War Office. The details are of the briefest, but it would seem that as Prince Henry was on his way from Cape Coast Castle to Madeira on board H.M.S. "Blonde," he succumbed on the 20th inst. He had borne the long and fatiguing journey fairly well from Mansu to the base hospital at Connor's Hill, and hopes of a speedy recovery were entertained. All nurses will feel the truest sympathy with Princess Beatrice and the Royal Family in their sudden and heavy sorrow.

### OVER-TRAINING.

A "happy medium" seems hard to find in nurse-training, for whilst strenuously opposing inadequate instruction we are not infrequently obliged to face the evils of the other extreme. We have always pointed out the absurdity of small hospitals insisting on a four years' curriculum, whilst acknowledging that a contract to spend the same period in the service of St. Thomas's Hospital is perfectly fair on the nursing staff, because the terms provide that part of the time may be spent in other institutions at the matron's discretion. Hence a varied education is secured to the nurses without danger of their aspiring to be students of cases rather than skilled and sympathetic nurses. The tendency of a too long training is, in injudicious hands, to impede a nurse's usefulness. Already many doctors express alarm at the false position which she sometimes takes between them and their patients. Loyalty to the doctor and faithful service to his patients should not be lost sight of in the interests of case-taking or diagnostic feats.

### AFTER-CARE OF THE INSANE.

By the kind permission of Mr. and Mrs. Alexander, a drawing-room meeting was held at Aubrey House on behalf of the After-care Association for Poor Persons discharged cured from Asylums. The chair was taken by the Rev. and Hon. E. C. Carr-Glyn, who spoke strongly of the need of such a society, and other speakers testified to its value. The vast number of persons discharged from asylums every year include many who require help during the convalescent stage, and who are often in great poverty and in delicate health.

### A QUESTION OF AGE.

The matter of age is always being discussed in some way or other in regard to nurses, and there is at present a general idea that, after forty or forty-five, employment is obtained with some difficulty in certain branches of nursing work. Doubtless much depends upon each individual woman, as fitness is better measured by personal capacity than by actual years. But the age question has other aspects, and at Yarmouth, for instance, it was thought by some people that a nurse of eighty-two ought not to be pensioned yet. She entered the service of the Board at sixty-two, and after twenty years' work one guardian is still of opinion that it is desirable for her to remain until she is entirely incapacitated! The comfort of the sick and infirm inmates does not appear, from the press report, to be considered at all by those who opposed the superannuation of this aged nurse, but probably further consideration may result in a juster view of the guardians' responsibilities to the poor and to the public who have modern ideas on the great age question.

### THE REGISTRATION OF NURSES.

It appears from a letter we have received that the Plaistow Maternity Charity, not the Glasgow Maternity Charity, was represented at the conference on the 14th inst., and that their representative voted against State registration. The speaker appeared to say "Glasgow," but several present thought he said "Gloucester," and this is how the mistake arose.

### A NURSES' HOME AT SHEFFIELD.

The new Home in connection with the Sheffield General Hospital will be a great boon to the nursing staff, who require better and increased accommodation. A suggestion has been made that the laying of the foundation-stone should take place when Her Majesty pays her promised visit to Sheffield this spring. It is, however, uncertain if the Queen will see fit to undertake that ceremony in addition to the opening of the new municipal buildings which she has graciously consented to do.

### DISTRIBUTION OF CERTIFICATES AT SUNDERLAND.

Certificates have been presented to the probationers who have completed three years' training at Sunderland Union Infirmary, and have passed an examination held by medical men unconnected with the infirmary. The success which has attended the introduction of systematic training at Sunderland Infirmary cannot fail to be gratifying to Dr. Prowde and Miss Cowan, to whom the results are largely due. They are both to be congratulated on the instruction by which they have qualified their pupils to satisfy outside examiners in general nursing, as well as to gain the diploma of the London Obstetrical Society. The awarding of the certificates was made the occasion of a pleasant gathering of friends and an excellent amateur concert.

### HELSINGFORS HOSPITAL.

The excellent condition of the hospital at Helsingfors shows that modern medical requirements are thoroughly understood in the Finnish capital. Women's work also seems to receive due acknowledgment, for it has just been announced that the degree of Doctor of Medicine has been conferred by the University upon Fraulein Eskolin, she being the first lady to whom this honour has been paid at Helsingfors.

### THE SERIOUS CHARGE AGAINST THREE GREAT HOSPITALS.

IT will be noticed, on reference to the verbatim report of the speech of Mrs. Bedford Fenwick at the conference held at the offices of the British Medical Association on the 13th inst., which we published in THE HOSPITAL Nursing Supplement, p. cxxxii., that it contains a most grievous charge against the managers of three important metropolitan hospitals. Alluding to registered nurses with three years' training, who hold certificates, Mrs. Bedford Fenwick said:—

"I find that these nurses, thoroughly competent and valuable servants of the public, go out and compete in the open market with probationers of one year's training from a variety of institutions, and, I regret to say, from the London Hospital, the Westminster Hospital, and, with little more training, from Guy's Hospital. As a rule, these institutions charge almost as much, or as much, as a thoroughly competent nurse would command. . . . Moreover, these institutions can afford to undersell the trained nurses. From an industrial point of view all this is bad."

If Mrs. Bedford Fenwick is correct in her statements it is much worse from a moral point of view, or, as Miss Wilson remarked, even from the "argument for the suffering patient," than from the industrial standpoint. But is it true? Mr. Burdett questioned the accuracy of Mrs. Bedford Fenwick's statements, and gave the grounds upon which he based his denial. The matter cannot, however, be allowed to rest here, for it is imperative that the authorities of the London, Westminster, and Guy's Hospitals should authoritatively admit or deny the facts stated. When contradicted, Mrs. Bedford Fenwick repeated her statements with emphasis, though she somewhat modified their form, and the medical men present were shocked by the charges in question. The London Hospital, and we believe the other two institutions concerned, advertise in the *Times* that "thoroughly trained nurses can be had immediately for all private cases," to which Mrs. Bedford Fenwick drew the attention of the conference. It is essential, therefore, that these hospitals should immediately inform the public whether the terms of advertisement are strictly complied with, or whether they send out to private persons who answer their advertisements not "thoroughly trained nurses" but probationers of one year's training only.

### NURSING AT CREDITON.

THE Crediton Sick Nurse Association had a very satisfactory report to lay before the annual meeting of subscribers, at which Lady Audrey Buller presided. The accounts show a balance in hand, and the work accomplished since the association's formation some ten years ago is altogether excellent.

### HEALTH MISSIONING IN BERLIN.

A SCHEME of a most useful character is in process of organisation at Berlin in connection with the proposed barrack sanatoria for consumptive patients. The plan referred to will be carried out by a committee of ladies, who are to devote themselves to the improvement of the hygienic environment of the families of these patients, doubtless with a view to benefit not only the former but also the surroundings of the latter when they return home. Such an undertaking will need to be carried out with as much consideration for the feelings of those visited as Miss Nightingale inculcates in the case of the health missioners who carry on a similar contest with insanitary surroundings in Buckinghamshire cottage homes.

### HOW NURSES CATCH TYPHOID.

IN a lecture before the National Health Society, Dr. Thorne Thorne incidentally mentioned one of the modes in which nurses catch typhoid fever—viz., by the nibbling of biscuits. It is worth bearing in mind, for nurses' work is long, and faint emptiness often comes on in the course of it, which they find by experience is relieved by a little food. The way that nurses sometimes catch typhoid fever is said by Dr Thorne Thorne to be from eating biscuits which they have kept in their pockets. However careful they may be in ablutions before fixed meals, the same precaution is not always taken before the hurried biscuit.

### A LOVED AND VALUED NURSE.

IT is with great regret that we note the sudden death, from heart disease, of Nurse Blair, a daughter of Dr. Blair, who was in practice for nearly fifty years in the parish of Dalry, in Ayrshire. Nurse Blair was educated in Edinburgh, and was a peculiarly clever, well-informed, and cultured lady. As soon as she was old enough, she entered the Western Infirmary, Glasgow, for training, and was afterwards staff nurse and then sister in the same hospital until a bad attack of rheumatic fever obliged her to return home to rest. As soon as she recovered her health she obtained the appointment of assistant matron at Cane Hill County Asylum, where she remained for over two years. Later on Nurse Blair joined the staff of the Nurses' Co-operation, New Cavendish, and during the last two years has been much valued by many patients, to whom she showed herself to be not only a clever nurse, but a true friend and good woman. Her death has been a great shock to a large circle of friends, amongst them to the matron of Cane Hill Asylum, whose guest she was at the time of her decease. After nursing a heavy case Miss Blair had consented to take a day or two's rest, and she was in excellent spirits and eager as usual to get back to work. Much sympathy is felt for her father by the fellow-nurses to whom she had greatly endeared herself. It was known that it was her earnest desire to die in harness, although no one anticipated her early death.

### SHORT ITEMS.

THE South Oxon Benefit Nursing Association held its annual general meeting at the Town Hall. The attendance was good and the report satisfactory.—The annual juvenile fancy dress ball in aid of the funds of the Victoria Children's Hospital, Hull, was a brilliant success.—An excellent report of the work of the Strabane District Nursing Society was laid before the annual meeting, at which the Duchess of Abercorn took the chair.—A large indoor fête has been held at Bedworth in aid of the funds of the nursing association.—The fifth annual meeting of the Buxton District Nursing Association was held recently, and the attendance showed the popularity and unsectarian character of the movement.—The Wisbech Guardians have raised the salary of their infirmary nurse from £25 to £30 in consideration of the responsible duties performed by her.—Dr. Patrick Campbell was last week presented with a handsome silver cigarette case by the members of the choir at Caterham Asylum. For many years Dr. Campbell has taken infinite pains in training the voices, whose proficiency well repays the time he has devoted to them.

### Lectures on Nursing.

By a Superintendent of Nurses.

### VI.—GENERAL CONDUCT.

### Causes of Irritation to Patients.

In walking about a ward or sick room study to move quietly and quickly. This is not at all difficult, and is merely a matter of habit. You may sometimes see a nurse lounging into her ward with her hands tucked into her apron pockets. Another one will come in post haste, not looking where she is going, perhaps run against someone going out, or she will knock the end of a bedstead and will shake a poor nervous, rheumatic patient, or will push against a table, making the crockery on it rattle, or will catch her dress on the knob of a door and tear it, causing people to shrink from her as from the proverbial bull in a china shop.

Then you get the dreamy nurse, who will walk into the ward kitchen with perhaps an earthenware teapot in her hand. Her eyes are fixed on some distant object, and she first knocks the spout against the door, then, suddenly roused, attempts to save it, in doing which she shakes it so violently that the lid comes off and breaks; or, if it happens to be on a tray, the whole thing falls to the ground with a horrid crash and the tray spins round. If this happens at night a whole ward may be roused with a sudden start, and the night's rest of several unhappy people be spoiled. This same individual, if told to hold a candle, will do it in such a way that the grease will gutter down into the candlestick, or drop on to the floor, or the flame will burn the hair of the doctor who is unfortunate enough to be examining a patient. If she takes a jug out of a basin, she will knock the side of the latter with it, and will probably hold it with one hand only; the whole strain being on the handle it will break off, and down will go the jug and the water swamp the floor. Then there is the mincing nurse, who enters the ward walking on tiptoe; if she has a door to open she will take nearly a minute to turn the handle; if a door creaks, she will prolong the agony by opening it as slowly as possible. She will then go to her patient (meaning to be very gentle) and speak in such a whisper that he has to ask her to repeat what she has said, and to lift his head in order to catch her words.

Speak distinctly to your patient; do not shout at him nor whisper, and do not stand behind him when you speak, or in his efforts to see you he will make his neck ache, and tire his eyes. Try to be perfectly natural in all you do. The sick are usually very inquisitive. Have you never heard people say of a deaf person—"when you do not want him to hear he hears fast enough"? and it seems as though this were the case with patients. They want to know all they can about their complaints, and they are suspicious; so, when you are whispering to their friends or others, they think your conversation must refer to them, hence their keen desire to hear all that is said. Especially should nurses be careful of what they say when patients are apparently unconscious. For instance, a lady now in India (and alive to tell the tale) had typhoid fever some years ago, and was exceedingly ill, and supposed to be unconscious; she heard the sister ask if it would be as well to telegraph to her friends, and the Doctor replied: "Oh, no, it is of no use, it can only be a question of an hour or two." Jerky speaking is very trying to the sick, so are sudden noises. How often I have felt a poor patient's pulse throb and the hand quiver as some thoughtless nurse has banged the ward door, or clumsily knocked something down, or even spoken in a sudden sharp way.

Try to make all about you restful; the presence of some nurses has this effect to a wonderful extent, and at most serious operations will impart confidence and courage to the poor sufferers, whilst, on the other hand, the presence of a thoughtless nurse distracts them.

### Light and Sympathy.

As a rule, you will find sick people turn their faces towards the light, and we know how cheering its effect is upon everyone. Notice, however, if the sun is streaming directly in on the eyes or head of your patient, and offer to pull the blind down far enough at least to protect him from the heat and discomfort of the direct rays. If there is fever, delirium, or head affection of any kind, it is best to keep the room dark, light being exciting. If your patient has had a restless night and seems inclined to sleep, he will stand a much better chance if there is darkness about him.

Now let me speak of something constantly lost sight of even by experienced nurses, and that is the importance of not only keeping the ward quiet at night, but of preventing talk and laughter in the kitchens adjoining the wards. I frequently hear nurses chattering in anything but quiet tones. Now, if your own mother were ill, and had just been "settled" for the night—if she had fallen into a nice sleep, would you not be very angry if two or three people stood laughing and talking just outside her bedroom door? You would call them exceedingly thoughtless. Well, this is constantly being done by those who in other respects are thoughtful and kind. Remember how important sleep is, how easily disturbed in the case of a sick person, and how difficult it is to sleep again if roused in the early part of the night. Remember, too, your mother may be ill, but may have no harassing home worries to disturb her. The poor found in hospitals are not usually so fortunate; they have all sorts of troubles to contend with. Some do not know if, when they get well, they may not find others filling their places in their so-called "homes"; some may dread finding their "little homes" broken up, their furniture, and even their clothes, pawned for drink; some may fear losing their situations and being turned adrift on the world, and their one thought is, "How can I get well most quickly?" I often marvel at the patience of our sick in not complaining of the talk too often carried on by nurses. Do let me urge you to be sympathetic! At the same time never "put on" sympathy; it is an affectation quickly detected by sufferers, and affectation of all kinds is intolerable to invalids. "All likings and aversions of the sick towards different people will be found," says one, "to resolve themselves into presence or absence of care in these things." Do not forget poor patients cannot complain, as rich ones very quickly do. Many a shy person will pass a bad night rather than speak to a nurse about some small yet great annoyance, such as a door ajar or window rattling.

### Economy.

I would press upon you the importance of studying economy, whether in a hospital or private nursing. Things are provided in such large quantities in a hospital that nurses are apt to forget that they cost money, and are frequently reckless in the use of them. Do not forget that an institution supported by voluntary contributions, if conducted on extravagant lines, is greatly curtailed in its usefulness, the number of people benefited being, of course, reduced. Learn to discriminate between stinginess and wastefulness.

Find out the value of materials used for dressings. It is most important that you should try to save expense. If, for example, you were nursing in the house of a poor professional man, where the illness of wife or child meant no little strain on a small income, how important it would be that you should avoid spending one penny unnecessarily, that the family might feel you were in every way a comfort and help. For instance, you might put on a clean sheet one morning, and dry and air and fold the one taken off. At night you could put this one on, reserving the one just removed for the next morning; the same might be done with the nightdress.

You would thus make the patient more comfortable (for change of linen is comforting), save washing, and save appearances, not making the comparative poverty too obvious by letting the patient lie in crumpled, dingy-looking linen.

In every hospital one hears complaints of the number of thermometers broken—and how often is this simply the result of carelessness? If asked how one got broken a nurse may say, "Oh! a patient broke it," and on asking which patient it may turn out to be a young child who could not possibly be expected to know the value of the piece of glass, or a man more or less delirious who may not be fully conscious of having to guard it, or the nurse may have left it so long under a patient's arm that he has dropped to sleep, and so the thermometer has got broken.

### Presentations.

Ladywell Sanatorium, Salford.—On the 17th instant the nursing staff of this institution presented Miss Thomas, the lady superintendent, with a solid silver afternoon tea set, with Doulton ware cups and saucers, on the occasion of her completing the twentieth year of service in connexion with the Salford Corporation hospitals. The present was accompanied by an address congratulating Miss Thomas, and hoping that she may be long spared to continue in so good and noble a work. We do not doubt that the Corporation also proposes to acknowledge in an adequate manner such faithful service, although the fact has not been yet made public.

In consequence of the recent appointment of an additional medical officer to the staff of the Poplar and Stepney Sick Asylum, Dr. E. M. Goldie has been transferred from the female to the male side of the asylum. The nursing staff on the female side have taken this opportunity to present him with a handsome silver-mounted whist case and a silver match box, as a token of their sincere regard and appreciation of his courtesy and work during the time he has had charge of that side of the asylum. The female patients have also testified their sense of the great kindness and careful treatment they have received at Mr. Goldie's hands by presenting him with a silver-mounted ivory paper knife.

### Novelties for Nurses.

### MESSRS. GARROULD'S WINTER SALE.

Proverbial are the bargains which are to be picked up during the month of January at the various sales held at establishments great and small in the metropolis. Not the least attractive of the many we have visited has been that of Messrs. Garrould. During the last few months considerable enlargement and rebuilding of the already extensive premises has taken place, and goods which had become slightly soiled or damaged during the operation are being offered at a marvellous reduction to the public. In materials and dress lengths the bargains are yet more noticeable; heavy serges and tweeds in black and navy blue can be had for 3s. 4½d. the full dress. This will, no doubt, appeal to our nurse readers who are constantly in want of these serviceable goods. Prints and Oxford shirtings are another item demanding attention. The colouring and designs in these are charming in every way, and they are guaranteed to wash well. In hosiery there is a large assortment; cashmere, cotton, and thread stockings are so reasonable that considering the wear and tear these articles are subjected to the inclination to stock oneself for the year is hard to resist. Linens and blankets, furniture, and carpets all form a part of the items of the extensive catalogue that has recently been issued. A visit to 150, Edgware Road, at the present season is one with which pleasure and profit will be equally combined.

Mr. H. K. Lewis, 136, Gower Street, has sent us a specimen-sheet of his admirable Nursing Chart, which cannot fail to commend itself to all private nurses, as well as to the managers of institutions where sick people are received.

### The Evolution of the Thermometer.

The unwisdom of the ancients and even of those who lived in the middle ages is a theme on which many of the unthinking sages of the nineteenth century are only too willing to descant. To those, however, who reflect it is an unending matter of surprise to find how far these giants of days gone by penetrated into the secrets of nature, entirely unprovided as they were with instruments of precision. It was indeed a matter of making bricks without straw. Looking at the laborious researches of recent years, it no doubt appears a mere matter for subordinates to investigate, with much labour and tedious care no doubt, but still with no striking exercise of genius, the melting points of various bodies. But how about the times when no thermometers existed, and when there were no means, beyond mere rule of thumb tricks of the laboratory, for comparing one temperature with another. In "Science Progress" for January we find an article on "The Evolution of the Thermometer," which shows how recent is the power of measuring temperatures with anything like accuracy. Even the knowledge that the melting point of ice and the boiling point of water were fixed points seems not to have been recognised until about the middle of the seventeenth century, when both Hooke and Boyle proposed the freezing point of water as zero. In regard to this it is interesting to find that at that time Boyle was persuaded that waters of different origins congealed at different temperatures. Yet the instruments of the day did not enable him to solve this doubt. In our admiration of uniformity we are apt to laugh at the Fahrenheit scale and compare it, much to its disadvantage, with the centigrade. But when Fahrenheit made his thermometer the centigrade did not exist even in men's minds. Such things as degrees were only thought of in regard to circles which were divided into quarters of 90 deg. each. We need not, then, be surprised that Fahrenheit began by making a +90 and a —90 on each side of a zero. The difficulty was to find a fixed point anywhere; but it being imagined that a mixture of ice and salt was the greatest cold attainable (!), that became his —90 deg., while +90 was "blood heat." By degrees, however, more easily recognised fixed points became available, and in 1714 Fahrenheit changed his zero to the lower of the points mentioned above, taking the freezing point of water as 8 deg., and blood heat as 24 deg.; and finally, about 1720, he divided these degrees into fourths, and thus we got the scale we now use, the freezing point standing at 32 deg., blood heat at 96 deg., and the boiling point of water at 212 deg. But the centigrade scale was not suggested until some time after this, and was not generally used in France till after the Revolution. Thus by gradual degrees was introduced an instrument of precision by which it became possible to compare and record temperatures, and by which science was enabled to make rapid strides. It is by accurate measurement that knowledge steps forward; and so far from wondering at the ignorance of the ancients, we should rather marvel at the general accuracy of the knowledge which they gained by sheer observations, unaided by mechanical means.

### The Midwife and the Midwifery Nurse.

We held over last week that portion of the discussion at the conference at the offices of the British Medical Association which was, perhaps, after all, of the most practical value to the progress of nursing. It was pointed out that the registration of women trained in general, medical, and surgical nursing was not a question which Parliament would be likely to take up, having regard to the fact that the register of the Royal British Nurses' Association is now open, under proper regulations, to every qualified nurse in the country who can become a registered nurse at any time she may wish to do so, and so secure any advantage which registration can afford. Undertakings incorporated by Royal Charter do not need to be re-incorporated by Act of Parliament, as such a step would be mere waste of the time of Parliament. The present proposals, therefore, to introduce a Bill for the State registration of nurses, when registration is now being properly conducted by a chartered corporation must (if they ever arrive at the Bill stage) secure prompt rejection by Parliament.

The true motives of the promoters of such a Bill must be looked for outside the question of registration of nurses altogether. One of them, no doubt, is that which Dr. Woodcock mentioned, *i.e.*, the desire to delay the passing of a measure for the registration of midwives. Another motive may be found in the domestic difficulties in the Royal British Nurses' Association, which it is an open secret are nearing the acute stage. We hope, therefore, that members of Parliament and all who take an impartial interest in the welfare of nurses, their education, training, and work, will not be misled if a few individuals persist in introducing a Bill for the State registration of nurses.

On the other hand, the question of the State registration of midwives, as medical practitioner after medical practitioner pointed out at the conference, is a most pressing and urgent one, which Parliament ought to deal with on its merits and settle without delay. Midwives are not nurses, and the question of the registration of midwives is quite distinct from, and necessarily entirely outside, the question of the registration of nurses. As Miss Wilson well said, "I protest against the term midwifery or obstetric nurse being applied to a woman who has charge of a woman in her confinement. Whether trained or untrained, she has charge of natural labour, and is not a nurse. . . . She is essentially a midwife, and the duties of the midwife and of the nurse are quite distinct." It is an injustice to apply the term "nurse" to any woman who has not had adequate training in medical, surgical, and other branches of nursing, and who holds no certificate of competency. Midwives, as a class, hold a certificate in midwifery, but they have had little or no experience in nursing, and are not trained or certificated nurses. The medical profession, as a learned profession, cannot under existing conditions permit anyone who purports to represent them to advocate in Parliament the introduction of the term "midwifery nurse" into any Act which its promoters may seek to pass. To do so would show lamentable ignorance, and an attitude towards the whole of the trained nurses in this country which would be unjust, and an abuse of the proper meaning of the words used.

Again, there is, as we have said, crying, urgent, paramount necessity for the passing of an Act of Parliament for the education and registration of midwives and for the enforcement of adequate penalties on all who wrongfully act as midwives, and so endanger the lives of the mothers of England. This question is one which affects the well-being and the vital interests of hundreds of thousands of the poorer classes throughout the country. It will be seen that this fact was realised by more than one speaker at the conference, and that there was a general feeling amongst the medical representatives present that the question of permitting the State registration of general, medical, and surgical nurses is not one which Parliament can concern itself with now or at any time unless the system of registration established by the Royal British Nurses' Association should bring about results which are wholly absent at the present time.

On the other hand, legislation for midwives, a thing wholly apart and distinct from the registration of nurses, is an urgent question, which the best interests of the people must compel Parliament to deal with adequately and properly.

It is interesting to point out in conclusion to those who drafted the resolution passed at the annual meeting of the British Medical Association which was submitted to the conference, that it does not include midwives, as it merely declares that "it is expedient that an Act of Parliament should as soon as possible be passed providing for the registration and education of medical, surgical, and obstetric nurses." The term "obstetric nurse" is only another name for the monthly nurse, who may or may not hold a certificate in midwifery, and neither obstetric nor monthly nurses, as a class, are midwives at all. Hence the chairman of the conference would have been perfectly justified in ruling out of order all the speakers who discussed the registration of midwives, because, as will be seen, the resolution at the annual meeting of the British Medical Association did not include midwives, a fact which seems to have been overlooked, both by Sir Walter Foster and other speakers. The following discussion at the conference on the questions raised above cannot fail to be of interest:—

THE DISCUSSION.

Dr. WOODCOCK (Manchester): I should like to ask, if this is the right time, whether it is thought that the registration of midwifery nurses and that of medical and surgical nurses should proceed at the same time and on the same lines? I notice in the memorandum of Dr. Bedford Fenwick, clause 6, that a trained nurse is defined as a person who attends on the sick or a woman in labour only under medical control or direction. Now a difficult question arises here. An ordinary medical or surgical nurse always attends under medical control. That is absolutely necessary, and I am sure we all are under a great obligation for the service rendered to us by trained nurses. I wish to say that, and I can see it is desirable for these nurses to be registered and have a legal status. It is within my knowledge that many persons who are opposed to legislation altogether are disposed to take up a favourable attitude in reference to the general registration of nurses, with the view of delaying legislation with regard to midwives, which I think is urgent. Attempts have been made to legislate mainly in the interest of the poor women of this country and their offspring, and no doubt it is the serious intention, if not of the Government at least of some private members of Parliament, to do something in this respect, and it appears to me that if we allow this wide question, which may be desirable from many points of view, to interfere with legislation which is absolutely necessary in the interests of the poor, we shall be making a great mistake. Then there is another question which largely affects the working classes. There are thousands of people in the poorer districts who cannot afford to pay for trained nurses, but who are waited on by women who possess common sense and a readiness to learn and carry out instructions. What do you propose to do with these? Instead of putting the medical and surgical and obstetric nurses together would it not be better to deal with the question of legislation for midwives, which is so badly needed, and leave the question of general registration over until that has been disposed of? No great harm is being done in postponing for a time a system for the general registration of nurses on behalf of whom Mrs. Bedford Fenwick has spoken so eloquently, and further consideration might lead to the development of some better system than has been devised up to the present.

Mr. BURDETT: Leaving for the moment the question of general registration I should like to point out that the phrase "a midwifery nurse" is an anomalous term which means practically nothing. If you take the practical position of midwifery nurses as understood now in this country you will see that they are confined to rural nursing associations, and that there is no such thing in practice as a midwifery nurse in any proper meaning of that term. The title midwifery nurse, if it means anything, implies a woman who is certificated and trained in midwifery, and also certificated and trained as a nurse. There are a few trained nurses who are also certificated midwives, but their number is so small as to be insignificant. Now, as a matter of fact, a midwifery nurse, so far as I can ascertain, and I believe I am perfectly correct in what I am saying, is a woman who has a certificate of the London Obstetrical Society or some certificate in midwifery, and who has had practically no training in nursing whatever. I venture to say that the medical profession, before they commit themselves to the term "midwifery nurse," should clearly understand how far it is practicable to find such a woman in this country at the present time, and what the term precisely means in the practice of nursing obtaining in this country at the present time. These are points which I do not think have been properly thrashed out, but the Lincolnshire Nursing Association is devoting its attention to them, and so I believe is the Queen's Jubilee Institute. It has been determined by some nursing associations to adopt the title of county nurse, meaning thereby a nurse who has a certificate as a midwife, and who has also had a few weeks or, perhaps, even six months' experience in some institution where in-patients are treated, so that she may have obtained a smattering of ordinary nursing in addition to possessing her certificate as a midwife. I think it is important to have a clear definition in dealing with this proposed term "midwifery nurse," and that it is essential that the medical profession should realise, as Parliament will promptly discover, that "midwifery nurse" cannot be accepted in any Act of Parliament, because there are practically no such workers to legislate for at the present time. There are midwives and monthly maternity or obstetric nurses, though the majority of the latter are not entitled to be called nurses, for they hold no certificate of training, nor are they trained as nurses.

Miss Wedgwood: I fully expected that Mr. Langton would have been here to have represented with me the Royal British Nurses' Association. As he is not, perhaps I might just say that any nurse who wishes to be registered can be registered on the register of the association I represent, and therefore any nurse unregistered is so by her own wish. The only difference between our arrangement and that proposed appears to be that what is proposed would be compulsory, while ours is not. I might add that we interfere with no training school or any other body.

Dr. Ward Cousins (chairman of the Council of the British Medical Association): Can any nurse be registered by your association?

Miss Wedgwood: Yes; subject to certain conditions, all nurses applying for registration, if found suitable for registration, are registered on payment of a fee.

Dr. Ward Cousins: I presume three years' training is necessary?

Miss Wedgwood: Yes; but not necessarily all the time in one hospital.

Dr. Ward Cousins: The question is, whether this meeting would not recommend our proceeding with some form of midwives' Bill, or midwifery nurses' Bill, and allow the important question of the general registration of nurses to stand over, in face of the Board, for the registration of nurses, which exists at the present time. The Royal British Nurses' Association, we are informed, will receive a nurse on their register who has been properly trained. I think that it is a great step in the right direction. And the next point is that Dr. Woodcock has placed most definitely before us, and I think all are willing to accept his proposition, that it is a crying necessity that something should be done in the interest of the poor of this great and Christian country in the direction of supplying what is absolutely essential—trained midwives.

Dr. Miller: I should like to say one word to express my hearty sympathy with the way in which this matter has been put by Dr. Woodcock and Dr. Cousins. The matter of proper training, education, and registration, and afterwards the control of women qualified to attend the poorer class is one that calls for legislation as early as possible. The other matter, that of the registration of general nurses, may be desirable, but it can afford to wait until the more important matter has been dealt with.

Dr. Woodcock: There is no immediate necessity for legislation on the general registration of nurses, but there is immediate necessity for legislation on the midwifery subject, and it is somewhat of a reproach to us as a profession that we have not been able to agree on some arrangement whereby something should be done in the interests of the poor of this country.

### Where to Go.

St. John Ambulance Lectures at the Polytechnic, Langham Place, London.—"First Aid," on Thursday afternoons, at 3.15, commencing 23rd inst. "Home and Sick Nursing," same afternoons, at 4.30.

Dr. Cotman is now lecturing at the offices of the Royal British Nurses' Association, 17, Old Cavendish Street, on Thursdays, at three p.m. The subject for January 30th will be "Epileptic, Hysteroid and other Convulsions." On Friday, the 31st, at the same offices, at eight p.m., Mr. Clinton Dent will give an illustrated lecture on "Mountain Myths and Legends." The public are admitted to both lectures on payment of 1s.

National Health Society.—A lecture was given before the National Health Society on January 21st by Professor Corfield on "Defective Drains and Sewer Air as Causes of Disease." He showed that exposure to sewer air was especially apt to lead to sore throat, and whether the diphtheria poison were contained in such air or not, persons exposed to it were at any rate predisposed to develop serious attacks of that disease.

Yachting Exhibition.—The fourth yachting exhibition, which is now open at the Royal Aquarium, is notable for the number of novelties on view, amongst which is a horseless carriage with petroleum motor, and a steam launch, driven by liquid fuel, which has been built especially for H.R.H. the Prince of Wales, to be carried on the deck of the "Britannia" when cruising, and will be used to tow the celebrated yacht to her starting point when racing. H.R.H. the Prince of Wales is understood to have signified his intention of seeing the launch at the Aquarium before it is put into the water.

Hygienic Education for Ladies.—A course of lectures by Dr. A. T. Schofield commenced at the Polytechnic, Langham Place, on January 21st, and will be continued each Tuesday afternoon. At the first lecture Dr. Schofield pointed out to his audience the simplicity of the study of the laws of health compared with the science of cure, emphasising the difference between lectures on simple hygienic principles, which should enter into the training of every woman, and nursing classes. He urged his hearers to do all in their power, by obeying the laws of hygiene, to die natural rather than premature deaths. If natural death might be likened to the stopping of a clock when run down, premature death would mean arrest of the pendulum. Life was now not only lengthened, but sweetened by better health, owing to our wise sanitary laws. Dr. Schofield pointed out that when he began the study of this subject some years ago, the average length of age in this country was 36 years; the limit had now extended to 44. A second lecture will be given on Tuesday next, the 28th inst., by Dr. Louis Parkes, on "Ventilation and Cubic Space in Houses," at 53, Berners Street, W., at five p.m.

### Minor Appointments.

West London Hospital.—Miss L. Gaved-Wills has been appointed Night Superintendent at the West London Hospital, Hammersmith. She was on the staff of the South Devon Hospital, Plymouth, for over eight years, where she also received her training. We wish her every success.

Nurse S. W. Doughty has been appointed Charge Nurse of the infirmary wing of the St. Pancras Workhouse. She received her training at the Norfolk and Norwich Hospital, having since been for one year charge nurse of the male wards at the Chichester Infirmary, and for three years nurse matron at the Fever Hospital, Southend-on-Sea. Nurse Doughty's testimonials are good, and her experience has been valuable. We hope success will attend her in her new post.

### Private Nursing.

By Sister Grace.

Qualifications Necessary.

No one who has the usual amount of common-sense would think of undertaking nursing in any of its branches without a fair share of good health, but people of less robust constitution than is necessary for continued hospital work may succeed very well as private nurses, because after the preliminary training of two or three years in the wards the work does not involve so much bodily fatigue. There is very little walking, fewer stairs, and comparatively little standing. The fatigues of private nursing are of an entirely different character. Unless the case is sufficiently serious to require two attendants, the nurse will frequently be on duty to a certain extent both day and night. All institutions have different rules in this matter; some matrons insist, very properly, upon the nurses having eight consecutive hours out of the twenty-four off duty, and if night work is being done in combination with day, every third night must be spent in bed.

But I earnestly caution every one who takes up the work of nursing not to "stand on her rights" in these matters. You must, of course, obey your superior's commands, and friends of patients are often exacting, but every matron will allow a good nurse a certain latitude; and, believe me, if you do your work in a cheerful, ungrudging spirit, and do not allow your patient to feel that you regard your work in a commercial spirit (so much time given for so much money received), your life will be happy and you will diffuse a bright and cheerful influence wherever you are placed. The life is often monotonous, and there is a great deal of spare time at the disposal of the nurse, so that it is well that she should be a person of resources that time may not hang heavy on her hands.

I need hardly say that high principle, good temper, tact, and unwearied patience are indispensable qualities in a private nurse.

London and Provincial Institutions.

Were I seeking a nurse myself I should feel it difficult to make a choice between London and the provinces, as there are advantages in each. Nurses attached to London institutions are more likely to keep up with all the exigencies of modern science because the doctors under whom they work are more "up to date" in their treatment than those who live in villages where provincial nurses are so much employed. On the other hand, nurses working in provincial institutions are better known individually to the superintendent, and there is less fear of getting undesirable people. In the hospital where I was sister many years we had a very successful and valuable private nursing staff of thirty-five, and I attribute a great deal of the popularity which our nurses undoubtedly enjoyed to the fact that our matron controlled both the hospital and private nurses, and that she never admitted strangers to her staff; they were all trained in the hospital, and were personally known to her. Thus a nurse who had passed through her hospital course of two or three years had been during this period under the influence of our matron, and no one was sent out who was not well known to us all. Naturally all were not of equal talent, but it was almost unknown to find one turn out unsatisfactory as to character. It was the custom, if these nurses were unemployed for a time, to send them into the wards to take special work, and sometimes night work, thereby enabling them to see something of the newer forms of treatment, and so to avoid the danger of becoming behind the times in their work. This plan worked admirably for them, but I think it was not advantageous to the hospital. As a natural consequence of working the two institutions together, the hospital was sometimes temporarily robbed of its trained probationers when there was pressure of work on the private staff. I believe this is unavoidable in provincial institutions worked on these lines, where the honorary medical staff consider themselves entitled to insist upon nurses being supplied to their own cases, even when there are none left in the nursing home. This causes a drain on the hospital staff of nurses, which those same doctors are the first to resent.

Let me warn you to be careful to what home you attach yourselves when your training is complete. There are many unprincipled people who make a living out of the earnings of the nurses who belong to their so-called homes (a cruel satire on the name of home), and if the nurse falls ill she is at once dismissed, as no longer representing capital. It is difficult always to recognise the true from the false, as these people manage to blind the public, and frequently head their papers with a committee and an imposing list of lady patronesses. Do not answer specious advertisements, nor join any home of which you know nothing, or that is not mentioned in "Burdett's Hospital Annual."

Training Specially Adapted for the Work.

I always asked my probationers what branch of the profession they wished to join, as one may give more profitable instruction if the nurse has decided on her future course. For private nursing it is very necessary to understand simple sick cookery in all its branches, and that you should know how to clean a grate and keep up a fire without noise, that you should understand all domestic work; no work is menial that conduces to the comfort of the patient. In many homes, through poverty, there are no servants; in others servants are plentiful, but the invalid is too ill to admit of their entering the sick room. Thus it is all important that the nurse should be well up in all these matters.

### Christmas Entertainments.

The sight of the Sheriff of London's gay coach outside the National Orthopædic Hospital on Saturday attracted a small crowd on the pavement. Within the wards the patients were meantime enjoying the novelty of seeing their Christmas gifts cut off the beautiful tree by the Sheriff (Mr. Cooper) himself. His lace ruffles and golden collar of office were greatly admired by the little patients, to whom the handsome livery and powdered heads of the Sheriff's two footmen were almost equally attractive. The latter helped in the distribution of the gifts, and so did most of the guests. The male patients were distinguished by crimson and black jackets of an excellent shape, and the children—well cared-for, happy-looking creatures—were very soon engrossed by their respective toys. Some handsome *Truth* dolls adorned the end of the pleasant ward in which the tree was placed, and carols were well sung by the patients.

The patients of the Carmarthen Infirmary spent a very happy afternoon and evening on the occasion of their annual treat. A liberal tea was provided, to which each patient was allowed to invite three friends. Afterwards a magnificent Christmas tree gladdened the hearts of old and young alike, for it was laden with beautiful presents suitable to all. In the large hall a concert afterwards took place, some very good songs being sung by the visitors. Some duets were sung by a little girl and boy, both suffering from hip disease. The proceedings terminated at nine o'clock with the National Anthem.

Christmas passed very pleasantly at Sunderland Union Infirmary, where an excellent tea was provided for the patients, followed by concerts in allthe wards. The children were also entertained with a magic lantern and Christmas tree. The nurses presented a handsome travelling clock to Miss Cowan, their superintendent, as a token of their esteem and gratitude.

### Matrons and "Private Practice" (!).

The danger of a little knowledge is proverbial, and when its exercise is encouraged by pride of office its evils are greater still. We need not attempt to hide the fact that one of the temptations before which too many nurses fall is that of using the smattering of medical knowledge which they have perforce obtained during their training to other purposes than that of helping them in their purely nursing work.

What happens is this. A cottage hospital is established, and a capable nurse-matron is appointed. Time, perhaps, hangs heavily for lack of interesting cases, and a certain amount of visiting intimacy arises between the nurse and the wives and families of those who form the committee of the hospital. Then comes the temptation to "show off," to give little hints about the baby, to suggest a tonic for the pale-faced daughter, and gradually, as far as may be, to supplant the family physician. Nothing is more easy or more insidious. It is not like calling in a new doctor and turning off the old one. Nothing of the sort is necessary. The committee and their wives all feel that they still can call in the doctor when they want him—but that for little ailments nurse is "very nice." So the evil grows, and a wholesale system of illegitimate practice is set up, for the nurse, having accommodated one member of the committee, cannot refuse another. To the laity this free "medical attendance" (!), rewarded as it may be by some cheap little present now and then, appears a charming substitute for doctors' bills, while to the nurse it is doubtless pleasant, just at first, to be asked to houses where she otherwise would not go, and to be flattered by the deference with which her pronouncements are received. Probably her own perception of the smallness of her knowledge and the vastness of their ignorance gives an added piquancy to the situation.

But in the end a Nemesis comes upon the partners in this ill-advised arrangement. Dissatisfaction soon arises among the wives because their babies do not all get seen immediately, then some case is sure before long to end in disaster, the hospital gets in bad odour with the doctors who sniff quackery and illegal practice, and with all outside the managing clique, who do not see why they should not share the good things; and the nurse, tired with her ill-spent days and her long rounds among her well-to-do "patients," thinks of her neglected hospital and district work, looks ruefully at her little collection of flashily-bound books and cheap bronzes, the easy substitutes for fees, and asks herself whether it is all worth while. If she is wise she resigns before she is hated, and goes off to a new sphere amid a salvo of testimonials, which after all do not cost much. But it is hard upon her successor. On her falls the evil consequences of the bad example. A bad nurse repeats the old game with the old result, but a good one does her proper nursing work, and if in an emergency she should go to see a patient she honestly advises what she knows to be best, viz., that a doctor should be sent for,—and is thought a fool for her pains. It is not long before she receives her dismissal as lacking in sympathy and unsuited to the place. Meanwhile the original cause of all the mischief has by dint of her beautiful testimonials got another appointment, and is sowing the seeds of the same evil in another village.

We have but two words to say on the subject. To the nurse we would say that any attempt to usurp the function of the doctor is not only ill-judged but actually dishonest; partly as being a matter of false pretence, partly as bringing into ill-repute an institution she is engaged to support and help. To the subscribers to any cottage hospital we would say that the acceptance by members of the committee of services from a nurse, who is paid to devote herself to the institution and its works, is a dishonesty of precisely the same kind as if these good committee-men walked out of the institutions with loaves and bandages and bottles of physic under their arms, and that the sooner such managers are dismissed from office the better.

### Funeral of an Army Nurse with Military Honours.

Last week, under the heading "Our Sister in India," we announced the death of Sister Evelyn Mary Leicester at Allahabad, and we are now able to give further particulars to her many friends in England. Sister Leicester first complained of feeling ill on December 21st, when she was at once seen by the surgeon-major and ordered to bed. Every attention was paid, and two nursing sisters devoted themselves to her, two officers' wives, her special friends, being the only visitors admitted. On the 25th she became much worse, and died very peacefully in the dawn of the new year. Sister Leicester was laid to rest the same day with full military honours. The procession consisted of a company of the Royal Irish Fusiliers, with arms reversed, then the drums and fifes of the Royal Irish Fusiliers, followed by a gun carriage, drawn by six horses, on which lay the coffin, covered with cream coloured drapery and buried in beautiful wreaths and crosses. A troop of the Royal Artillery marched behind the gun-carriage, and after them came the general and his staff, officers of the 1st R.I. Fusiliers, Royal Artillery, 8th Bengal Cavalry, 5th Bengal Light Infantry, Staff Corps, Army Medical Staff and Departments, several civilian gentlemen, and the nursing sisters and hospital orderlies, carrying more wreaths. The drums and fifes played the "Dead March," and on reaching the cemetery the band played Chopin's "Funeral March." Surgeon-Major Williamson, A.M.S., Surgeon-Major Nicholson, A.M.S., Surgeon-Captain Wilson, A.M.S., and Surgeon-Captain Nicholls, A.M.S., carried the coffin from the cemetery gate to the grave. The troops were drawn up in double line facing the grave, the drums and fifes behind them. The chaplain stood at the foot of the grave, the four A.M.S. officers on either side. The general and officers stood immediately behind the chaplain, and the two sisters facing them. At the conclusion of a most impressive service three volleys were fired over the grave. Many kind communications have been received from India testifying to the affectionate regard in which Her Majesty's nursing sister was held by rich and poor alike, and a little drummer boy's spontaneous "I am so sorry; she was such a nice lady," spoken in a broken-hearted voice at the funeral, found a ready echo in other loving hearts.

### Ophthalmic Hospital, Moorfields.

An enjoyable little dance was given by Mr. Newstead, the secretary of the Moorfields Ophthalmic Hospital, to the nursing staff on Wednesday, January 15th, in accordance with his yearly custom at Christmas time. The sisters and nurses each invited a certain number of friends, and the out-patient hall, where the patients' concert was held the week before, was prettily decorated with flags, and with its polished floor lent itself well for the occasion. Hospital parties need to be early ones, or the work next morning might suffer, so dancing was from half-past seven to twelve o'clock. Refreshments were served in the museum and library. Mr. Newstead and Miss Robinson were indefatigable in looking after their guests, and a pleasant evening was spent by all who were present.

### Everybody's Opinion.

[Correspondence on all subjects is invited, but we cannot in any way be responsible for the opinions expressed by our correspondents. No communications can be entertained if the name and address of the correspondent is not given, or unless one side of the paper only be written on.]

### NURSES AND LAY COMMITTEES.

"CRITERION" writes: In your issue of the 11th inst. you comment on some district nurses being "managed" by lay committees. I am quite of opinion that such a state of matters prevails to a great extent in country districts. I know of one parish where a "Queen's" nurse is managed by a committee of ladies, including the wives of most of the Dissenting ministers. This committee meets once a week, in the apartments of the nurse, and amongst other business transacted the visiting book, kept by the nurse, is carefully scrutinised, to ascertain the number of cases she may be attending at the time. Should there be but few on her book she is requested to call at any house in which she may, casually, learn there is illness, and offer her services, and this without the knowledge or sanction of the doctor attending the case. The nurse is further instructed to offer her services and supply medicaments and nourishment to poor people, who are in receipt of outdoor parochial relief, thus saving the pockets of the ratepayers and the medical officer of the parish, at the expense of the subscribers to the Nursing Association. Naturally doctors are chary of having anything to do with a district nurse under such circumstances, more especially as the nurse enters in her book the nature of the malady from which the patient suffers, as well as the treatment pursued in the case, thereby making it almost public property. By reason of the methods pursued by the committee of the nursing association in question, many patients object to the nurse attending to them, and I consider they are quite justified in their objection. Unless some radical change is effected in the working of district nursing associations, I am afraid that the movement will become unpopular.

[We sympathise with our correspondent, and consider no district nurse should be encouraged to visit any doctor's patients without first consulting his wishes in the matter. We find, as a rule, that when this is done the medical man is only too glad to accept the aid of a competent nurse. As regards the "treatment" being "booked," does not this refer merely to formal notes as to the nursing of the cases, the application, &c., which the nurse is instructed to apply?—ED. *T. H.*]

### THE PORTRAITS OF T.R.H. THE PRINCE AND PRINCESS OF WALES.

"SCOTTIE" writes from Edinburgh: Thanks a thousand times for the beautiful portrait you have given us of our beloved Prince in the Christmas number of THE HOSPITAL. It is, I am told by our very best portrait painter, the finest likness of him he has seen, and, if I may express myself so he just looks fresh and healthy, and a Prince every inch of him. The medical and surgical nurses throughout the length and breadth of the land owe you a debt of gratitude undoubtedly for giving them such a cheap and splendid portrait of their Royal patron, also for your enterprise and courage in venturing to publish it at such a price. I am afraid it won't pay you, but I hope it will. I feel certain that if two or three millions of portraits of our bonnie Princess and beloved Prince were issued just the same as you have given us it would pay the publisher well, and they would be bought up at once if put in reach of the general public, who have never got anything to equal them yet at a moderate price, and it would be a boon to poor but loyal people. I was away down in Ayrshire applying massage to a gouty gentleman when the Christmas number of THE HOSPITAL was delivered at my house in Edinburgh; it was forwarded to me. When my patient saw the portrait it contained he grabbed it and stuck to it too, and told me I might get another where I would, but he would certainly keep that one, and get one of our Princess if possible. I wrote up to my newsagent to get me a dozen or two of both; he succeeded in getting me a few, but not half as many as I wished for. I sent them all as New Year's gifts to my friends, except the two I kept and got framed for myself, and have now hung up in my room, and a bonnier pair of portraits are not in Edinburgh. When I look at them, as they hang now, I do wish I was rich enough to spare fifty guineas. Mr. Drummond Young would have an order from me to-morrow to paint them in oil, just as they are. That day may yet come when, perhaps, they may be King and Queen; but I hope in my heart they may remain our Prince and Princess for many years to come; and I also hope the day is not far distant when we will have the happiness of seeing them both in Auld Reekie. I am now fifty years of age, and I never had the pleasure of seeing a member of the Royal Family; but for all that, I know and feel I have them for my happiness, prosperity, and protection, as I told an American lady the other day, as sure as I knew and felt I had a bone in my finger. May God bless them all, from our gracious Queen to the wee baby York, is the prayer of yours gratefully.

### FRENCH SCHOOLS FOR TRAINED NURSES.

"A MENTAL NURSE" writes: Will you allow me to correct a few remarks in an article in THE HOSPITAL of January 4th about St. Anne's Asylum, Paris, as I can justly do, having been a nurse there myself. First about the low rate of pay, it must be remembered that we all have, in addition, our trousseau—and surely that counts for a good deal. If a nurse's conduct is good and she shows aptitude she has always a chance of being made a "first class nurse," and that means an increase of ten francs every alternate month. Secondly, about there being no distinction in dress, there is a distinction made, though it is not so evident as in hospital uniforms. The material of which the dress of a charge nurse is composed, and also that of an under charge nurse, is much finer than that worn by the ordinary nurse. The material is given to the first two classes, whereas the ordinary nurses receive dresses that more often than not do not fit them. The main distinction is shown in the caps; those of the charge nurses are entirely of black silk with silk and ribbons hanging down the back. Under charge nurses wear black silk caps with a white frill in the front; first class nurse, a white cap; and ordinary nurse, white cap also, but of inferior quality. As to the food, the description is quite correct. I have never seen, anywhere, nurses and patients better fed, and some of the public institutions in England ought to follow up the example given in France. Nothing such as your ex-nurse recently wrote about happens over there. Let me testify that what she says is quite true about food. Here I have been over two years in an English county asylum, and I think the only remedy for the present state of things would be a paid cook for the nurses only. Let the medical staff and officers have one for themselves by all means. I am now in a workhouse where our cooking is done anyhow by a pauper, and we have no puddings at all. Nurses that are on duty get no nights off duty during three months, but are allowed a half day a week, so they take this off their own allowances of sleep and go out, coming back in the evening to be on duty all night!

### HOSPITAL AND DISPENSARY, NEWARK-UPON-TRENT.

THE SECRETARY of the above institution writes: May I call your attention to an announcement in the book entitled "How to Become a Nurse," by Morten, where it states that probationers of the Newark Hospital must be members of the Church of England. Will you please note that no such condition is required at this hospital.

### For Reading to the Sick.

### THE WAY OF JOY.

**Motto.**

"THOU has proved that purest joy is Duty."—*H. Coleridge.*

**Verses.**

Nor hath thy knowledge of adversity
Robbed thee of any faith in happiness;
But rather cleared thine inner eyes to see
How many simple ways there are to bless.
—*Lowell.*

For he, and he only, with wisdom is blest,
Who, gathering true pleasures wherever they grow,
Looks up in all places, for joy or for rest,
To the Fountain whence Time and Eternity flow.
—*Wordsworth.*

I thank Thee more that all our joy
Is touched with pain;
That shadows fall on brightest hours,
That thorns remain;
So that earth's bliss may be our guide,
And not our chain.

For Thou, Who knowest, Lord, how soon
Our weak heart clings,
Hast given us joys, tender and true,
But all with wings—
So that we see, gleaming on high,
Diviner things. —*A. Proctor.*

**Reading.**

"When, therefore, spiritual comfort is given thee from God, receive it with thanksgiving; but understand that it is the gift of God, not thy deserving. Be not puffed up, be not too joyful nor vainly presumptuous; but rather be more humble for that gift, more wary too, and fearful in all thine actions; for that hour will pass away, and temptations will follow. . . .

"What art Thou to those who love Thee? What to those who serve Thee with their whole heart? Truly unspeakable is the sweetness of contemplating Thee, which Thou bestowest on them that love Thee."—*Thomas à Kempis.*

No man can say what mysteries of the yet unopened future are hidden in the picture of the mystic City; but if that City represent, as I have said, the glorified humanity, then there is much of it that we can understand already. It declares that the perfect life of man will be perfect on every side. One token of its perfectness will be its symmetry. . . . I hope that we are all striving and praying now that we may come to some symmetrical completeness. This is the glory of life. Do not dare to live without some clear intention toward which your living shall be bent. Mean to be something with all your might. Do not add act to act and day to day in perfect thoughtlessness, never asking yourself whither the growing line is leading. But at the same time do not dare to be so absorbed in your own life, so wrapped up in listening to the sound of your own hurrying wheels, that all this vast pathetic music made up of mingled joy and sorrow of your fellow-men shall not find out your heart and claim it, and make you rejoice to give yourself for them. And yet all the while keep the upward windows open. Do not dare think that a child of God can worthily work out his career or worthily serve God's other children unless he does both in the love and fear of God the Father. Be sure that ambition and charity will both grow mean unless they are both inspired and exalted by religion. Energy, love, and faith, these make the perfect man. And Christ, who is the perfectness of all of them, gives them all three to any who give up themselves to Him. . . . There, in redeemed and glorified human nature, is the true Heavenly Jerusalem.—*Phillips Brooks.*

### Notes and Queries.

The contents of the Editor's Letter-box have now reached such unwieldy proportions that it has become necessary to establish a hard and fast rule regarding Answers to Correspondents. In future, all questions requiring replies will continue to be answered in this column without any fee. If an answer is required by letter, a fee of half-a-crown must be enclosed with the note containing the enquiry. We are always pleased to help our numerous correspondents to the fullest extent, and we can trust them to sympathise in the overwhelming amount of writing which makes the new rules a necessity. Every communication must be accompanied by the writer's name and address, otherwise it will receive no attention.

**Queries.**

(111) *Cottage Hospitals.*—Can you tell me where to obtain an inexpensive work on Cottage Hospitals?—*J. N.*

(112) *Incurable Homes.*—Can you supply me with the names and addresses of homes where incurable cases are received?—*Paralysed.*

(113) *Dismissal.*—Can a committee dismiss a nurse without giving a reason, and without proof of her inefficiency? And can she claim legal compensation for damage to professional reputation by wrongful dismissal, &c., &c.?—*M. B. N. B.*

(114) *Correspondence Classes.*—What address should I write to respecting Miss Lamport's correspondence classes?—*A. W. and L. N.*

(115) *Armlet.*—When can I obtain the Princess of Wales' armlet worn by the nurses of the Royal National Pension Fund for Nurses?—*No. 10.*

(116) *Varicose Ulcers.*—Will any of your readers give me the benefit of their practical experience in dealing with varicose ulcers?—*P. M.*

(117) *Training.*—Can you tell me if there is an institution where I could be thoroughly trained as nurse and receive a salary? I have just arrived from Queensland, and have been in hospitals three years. I have good testimonials from small bush hospitals where no regular training was given. I am a widow, aged 24. I took THE HOSPITAL paper for three years in Australia. I often derived much instruction from it. If you can help me I should be much obliged.—*C. D.*

(118) *Masseuse.*—Please tell me the address of the Indian Nursing Service. Also kindly tell me if a trained masseuse could get her passage paid to India and get work there.—*Vera.*

(119) *Height.*—Can you tell me whether my height, which is only five feet, would prevent my getting admitted to a hospital?—*A. L. B.*

(120) *Shoes.*—Where can I get the Sister Eva ward shoe?—*M. C. W.*

(121) *Probationer.*—Do any of the larger London hospitals admit probationers at 21 years of age, and would a year's training at a provincial hospital disqualify me from entering a London hospital as a probationer?—*N. C.*

(122) *Testimonials.*—Where can I get testimonials printed at a reasonable rate?—*E. M. A. S.*

**Answers.**

(111) *Cottage Hospitals* (*J. N.*).—The third edition of "Cottage Hospitals" is obtainable from the Scientific Press for 10s. 6d.

(112) *Incurable Homes* (*Paralysed*).—Get "Burdett's Hospital and Charities Annual" from the Scientific Press, 428, Strand. In it you will find all particulars of the institutions about which you ask.

(113) *Dismissal* (*M. B. N. B.*).—In reply to M. B. N. B. it does not appear that the nurse can do much to defend herself. A committee properly elected has the entire responsibility for the management of the hospital, and in regard to matron, nurses, and servants, stands in the position of employer. No obligation would seem to lie on the committee even to give to a nurse any reason for her dismissal, and if they give the proper legal notice we do not see what she can do. The affair is quite otherwise, however, in regard to the subscribers to the hospital. No man's interest in an institution should be limited to the mere paying of his subscription, and if he sees that the hospital is being badly managed, or being managed by the committee for their own personal profit, he ought to agitate for a complete change of management. It must be remembered that the committee are directly responsible to the subscribers for the proper conduct of the institution, and this they should be made to understand by the aid of a little dose of publicity thrown upon their proceedings. In regard to this it may be well to bear in mind that if the nurse were so ill advised as not only to give medical advice, but actually to supply medicine from the hospital dispensary, to these committee-men, they would clearly be participating in a theft, and could be made amenable to law, which would be a pretty kettle o' fish in a little town!

(114) *Correspondence Classes* (*A. W. and L. N.*).—Write to Miss Lamport, 52, St. John's Wood Road, N.W.

(115) *Armlet* (*No. 10*).—They have been obtainable since September. In all cases when information is required apply to the Secretary, at the Offices of the Fund, 28, Finsbury Pavement, E.C.

(116) *Varicose Ulcers* (*P. M.*)—We strongly advise you to apply to a competent medical man; it is most dangerous to take the advice of all and sundry advisers.

(117) *Training* (*C. D.*).—Yes. You had better apply to matrons of some of the hospitals, of which you will find a list in "How to Become a Nurse," published by the Scientific Press, 428, Strand, London. We are very glad you have taken so much interest in THE HOSPITAL.

(118) *Masseuse* (*Vera*).—The India Office, St. James's Park. If your friend has had three years' training in general nursing as well as being a competent masseuse, she might get an appointment. We have never heard of any society willing to pay the passage of a masseuse who is not also a nurse.

(119) *Height* (*A. L. B.*).—If you are thought too short for some hospitals, others would take you, supposing you are suited for a nurse. Write and ask the Infirmary Nursing Association, 6, Adam Street, Strand, or some of the good provincial hospitals. It is always best to write straight to the matron at the institution, asking for rules, and enclosing stamped envelope for reply.

(120) *Ward Shoe* (*M. C. W.*).—We do not know this shoe. The S. Mary, sold by Garrould, Edgware Road, you may mean.

(121) *Probationer* (*N. C.*).—Twenty-five years is the age at which probationers are admitted into the larger London general hospitals. You might apply at some of the children's hospitals where younger candidates are accepted. You will find all particulars in "How to Become a Nurse," which you can get from the Scientific Press, 428, Strand. Previous training is no disqualification.

(122) *Testimonials* (*E. M. A. S.*).—The Scientific Press, 428, Strand, undertakes the printing of testimonials, reports, &c., at most reasonable rates.

